# Translation and validation of the German version of the Young Spine Questionnaire

**DOI:** 10.1186/s12887-021-02804-y

**Published:** 2021-08-24

**Authors:** Luana Nyirö, Tobias Potthoff, Mette Hobaek Siegenthaler, Fabienne Riner, Petra Schweinhardt, Brigitte Wirth

**Affiliations:** 1 Integrative Spinal Research Group, Department of Chiropractic Medicine, Balgrist University Hospital and University of Zurich, Forchstr. 340, 8008 Zurich, Switzerland; 2 Holbeinpraxis, Holbeinstrasse 65, 4051 Basel, Switzerland; 3 Winterthur Institute of Health Economics, School of Management and Law, University of Applied Sciences, Gertrudstr. 15, 8400 Winterthur, Switzerland

**Keywords:** Adolescence, Back pain, Childhood, Neck pain, Reliability, Responsiveness, Validity, Young Spine Questionnaire

## Abstract

**Background:**

Back pain in childhood and adolescence increases the risk for back pain in adulthood, but validated assessment tools are scarce. The aim of this study was to validate the Young Spine Questionnaire (YSQ) in a German version (G-YSQ) in children and adolescents.

**Methods:**

Children and adolescents between 10 and 16 years (*N* = 240, 166 females, mean age = 13.05 ± 1.70 years), recruited in chiropractic practices and schools, completed the G-YSQ (translated according to scientific guidelines) and the KIDSCREEN-10 (assessing health-related quality of life) at three time points. Test-retest reliability was determined calculating intraclass correlation coefficients [ICC_(3,1)_] using start and two week-data. Construct validity was investigated testing a priori hypotheses. To assess responsiveness, the patients additionally filled in the Patient Global Impression of Change (PGIC) after three months and the area under the curve (AUC) of receiver operating curves was calculated.

**Results:**

The ICC_(3,1)_ was 0.88 for pain intensity and pain frequency, indicating good reliability, 0.68 for week prevalence and 0.60 for point prevalence, indicating moderate reliability. Pain intensity, frequency and prevalence differed between patients and controls (*p* < 0.001) and, except point prevalence, between older (> 12 years) and younger control participants (*p* < 0.01). Health-related quality of life of participants with severe pain (in one or several spinal regions) was lower (KIDSCREEN-10, total score: F(4,230) = 7.26, *p* < 0.001; KIDSCREEN-10, self-rated general health: H(4) = 51.94, *p* < 0.001) than that of participants without pain or with moderate pain in one spinal region. Thus, altogether these findings indicate construct validity of the G-YSQ. The AUC was 0.69 (95 % CI = 0.57–0.82) and 0.67 (95 % CI = 0.54–0.80) for week and point prevalence, respectively, indicating insufficient responsiveness of the G-YSQ.

**Conclusions:**

Apart from the question on point prevalence, construct validity and sufficient test-retest reliability was shown for the G-YSQ. However, its responsiveness needs to be improved, possibly by asking for pain frequency during the last week instead of (dichotomous) week prevalence.

**Trial registration:**

ClinicalTrials.gov, NCT02955342, registered 07/09/2016, https://clinicaltrials.gov/ct2/results?cond=&term=NCT02955342&cntry=CH&state=&city=Zurich&dist=.

**Supplementary Information:**

The online version contains supplementary material available at 10.1186/s12887-021-02804-y.

## Background

Back pain and neck pain are leading causes for years lived with disability [1] and have a large impact on individuals, their families, employers and healthcare systems [2]. Spinal pain starts early in life and its prevalence increases with age, in particular around the age of 12 to 15 years [3, 4]. Back pain in childhood and adolescence is a significant risk factor for developing back pain in adulthood: the number of days in a given year with low back pain (LBP) in adolescence was shown to be associated with the risk of developing LBP in adulthood [5]. However, research on spinal pain in childhood and adolescence shows large heterogeneity in assessing pain prevalence, pain intensity and associated disability [3], and validated assessment tools are scarce [6].

In 2013, the Young Spine Questionnaire (YSQ) was developed in Denmark as an instrument to measure spinal pain in the young population [7], though it has so far only been tested for content validity in preliminary versions during the developmental process in a population of Danish schoolchildren in the age range of 9–11 years [7]. To allow for the practical implementation of the YSQ, it must be shown to provide accurate, valid and interpretable data. Therefore, the assessment of instrument validity and reliability is essential [8, 9]. Because the YSQ was originally not designed for capturing change, it is not known whether it is suitable to measure change in a longitudinal study design [7].

Thus, the first aim of this study was to translate the YSG into German (G-YSQ) to obtain a standardized assessment tool of child and adolescent back and neck pain which can be used in Switzerland (and other German-speaking countries). The second aim was to test the G-YSQ for validity, reliability and responsiveness in children and adolescents between 10 and 16 years.

## Materials and methods

### Translation

**Fig. 1:**
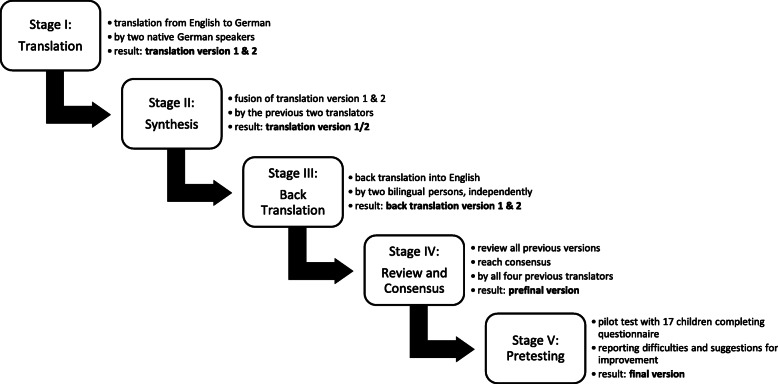
The five steps in translating the English Young Spine Questionnaire into the German version according to procedures recommended by Beaton et al. [10]

After authorization by the original authors of the YSQ, the questionnaire was translated into German in five steps according to the guidelines by Beaton et al. [10] (Fig. 1).

Two native German speakers (two researchers) independently translated the questionnaire forward from English to German (stage I) and produced one German version (stage II). Two bilingual (English and German) individuals (two researchers) independently translated this German version back to English (stage III). From these two versions, one common prefinal version was produced by the four forward or back translators (stage IV), which was then pilot tested (stage V) with 17 children and adolescents between 9 and 15 years (mean age = 12.25 ± 1.70 years). They were asked to report any difficulties in completing the questionnaire on their own and to make suggestions for improvement if considered necessary.

During the translation process, two cultural adaptations were made to the G-YSQ compared to the YSQ: (i) the terms ‘chiropractor’ and ‘physical therapist’ were omitted, because it was questioned whether Swiss children are familiar with the chiropractic profession and because there is no direct access to physiotherapy in Switzerland; (ii), the terms ‘stepfather’ and ‘stepmother’ were omitted, because these terms are uncommon in contemporary Swiss German. During the pilot testing of the prefinal version of the G-YSQ, three adolescents (aged 13 and 15) reported that they would prefer text or scales instead of faces indicating pain intensity but none of the children and adolescents reported any difficulties in completing the questionnaire. Nevertheless, the statements of several participants during the main study indicated the necessity for an answer option ‘I don’t know’ in the questions about paternal back problems.

### Study participants

Patients between 10 and 16 years of age with neck or back pain were recruited in seven private outpatient chiropractic clinics. The majority (76/100) came from one clinic specialized in the treatment of children and adolescents. Age- and gender-matched pupils (frequency matching) were recruited in four primary and secondary schools: After agreement of the teacher, two researchers from the Balgrist University Hospital, Zurich, Switzerland (FR, BW) personally informed the pupils about the study during a school lesson and distributed the information sheets. The study was approved by the ethics commission of the Canton of Zürich, Switzerland (BASEC-Nr 2016_00568) and was registered at ClinicalTrials.gov (NCT02955342). According to Swiss law, written informed consent of adolescents older than 14 years and their parents/legal guardians was required for participation. For participants between 10 and 14 years, oral informed consent of the children and adolescents and written informed consent of their parents/legal guardians was required.

### Outcomes

There were three measurement time points: at enrolment (start), at two weeks, and at three months after start. The patients filled in the questionnaires at the start in the chiropractic clinics and received the two-week and three-month questionnaires by mail from the coordinating researchers at the Balgrist (FR, BW). To acquire follow-up data of the control participants, the same researchers physically distributed the questionnaires at start, after two weeks and three months at the schools, mostly during a lesson in physical education. Both groups answered the questionnaires at all time points in paper form. The questionnaires consisted of the G-YSQ and the KIDSCREEN-10: the YSQ/G-YSQ assesses pain frequency, week pain prevalence, point pain prevalence and pain intensity (revised Faces Pain Scale (rFPS) [11]) in each of the three spinal regions (each visualized in a drawing) and asks about pain-related consequences (school absenteeism, activity restrictions in sports and care seeking behavior) as well as parental back problems and related work absenteeism [7]. The KIDSCREEN-10 [12, 13], including ten questions on physical and emotional well-being and one question on self-rating of general health, was used to estimate the impact of spinal pain on health-related quality of life (HRQoL). After two weeks, the control participants additionally answered a question on whether the state of their back problem remained stable during the past two weeks, and the patients completed the Patients’ Global Impression of Change (PGIC), which evaluates the patients’ rating of overall improvement on a seven-point Likert scale [14]. For the present study, PGIC was dichotomized (improved=’very much better’ or ‘much better’) [15, 16]. The assessment after three months included the G-YSQ, the KIDSCREEN-10 and the PGIC (patients only).

### Data analysis

Several participants reported spinal pain in the question on pain frequency, but reported a pain intensity of zero. This was observed for all spinal regions (neck: *N* = 8; midback: *N* = 7; low back: *N* = 8). Incompletely filled in questionnaires were included in all analyses for which they provided values. Pupils who previously sought medical care for back or neck pain were analyzed in the patient group because their care-seeking due to back or neck pain was considered an indicator for a back or neck problem. The G-YSQ data was analyzed by calculating sum scores across the three spinal regions for pain frequency (0 = no pain; 1 = yes, once in a while; 2 = yes, once or twice; 3 = yes, often), pain prevalence (0 = no, 1 = yes) and pain intensity (first face/no pain = 0, sixth face/very much pain = 5), resulting in a pain frequency sum score (range 0 to 9), a week prevalence and point prevalence sum score (each ranging from 0 to 3) and a pain intensity sum score (range 0 to 15). The sum score of the KIDSCREEN-10 was calculated according to the KIDSCREEN handbook [12].

### Validity

Construct validity was investigated using the start data by testing a priori hypotheses [17, 18]: (i) The sum scores of pain intensity, frequency and prevalence (week, point) are higher in patients than in controls; (ii) The sum scores of pain intensity, frequency and prevalence (week, point) are higher in older (> 12 [19–21]) than in younger controls; (iii) Self-rating of general health via the KIDSCREEN-10 significantly differs between patients and controls and strongly correlates (r_S_>0.6) with the sum scores of pain intensity, frequency and prevalence; (iv) The KIDSCREEN-10 total score moderately correlates (r_S_>0.4) with the sum scores of week and point prevalence (all measures refer to the last week); (v) Participants with severe pain report low general health and low total scores on the KIDSCREEN-10. To test this hypothesis, the participants were sub-grouped based on pain severity, defined by pain frequency and intensity, across spinal regions [22]. The resulting three severity levels ‘no pain’ (frequency: never, once or twice or once in a while; intensity: lowest two intensities on the rFPS), ‘severe pain’ (frequency: once in a while or often; intensity: highest three intensities on the rFPS) and ‘moderate pain’ (in between) were combined to an ‘overall spinal pain composite variable’ [22] with five levels: ‘multiple severe pain’ (‘severe pain’ in two or three spinal regions), ‘one-sited severe pain’ (‘severe pain’ in one spinal region), ‘multiple moderate pain’ (‘moderate pain’ in two or three spinal regions), ‘one-sited moderate pain’ (‘moderate pain’ in one spinal region), and ‘no pain’ (‘no pain’ in all spinal regions). If pain severity differed between the three spinal regions, the region with the most severe pain was used for the overall spinal composite variable. The hypotheses (i) and (ii) were tested using Mann-Whitney U-tests, hypothesis (iii) using Mann-Whitney U-test and Spearman correlation, and hypothesis (iv) was tested using Spearman correlation. Spearman’s coefficient values (r_S_) were interpreted as excellent (> 0.9), good (0.7–0.9), moderate (0.5–0.69), fair (0.2–0.49), or minimal to absent (0.0–0.19) [23]. Hypothesis (v) was tested using a one-way ANOVA (KIDSCREEN-10 total score) and Kruskal Wallis test (self-rated general health) and post-hoc tests in case of significance, between the five levels of the ‘overall spinal pain composite variable’.

### Reliability

Test–retest reliability was assessed based on the agreement between start data and data after two weeks. For the reliability of week and point prevalence, the data of those children and adolescents who reported no or stable spinal pain was used. Unweighted Cohen‘s Kappa and Intraclass correlation (ICC)_(3,1)_ was used to analyze categorical and ordinal data, respectively [17]. Kappa values were interpreted as 0.01–0.20 none to slight, 0.21–0.40 fair, 0.41–0.60 moderate, 0.61–0.80 substantial, and 0.81–1.00 almost perfect [24, 25]. ICC-values > 0.90 were considered excellent, 0.75–0.90 good, 0.50–0.74 moderate and < 0.50 as poor [26].

### Responsiveness

To test responsiveness, the area under the curve (AUC) of the receiver operating characteristic (ROC) curve was calculated as a measure to discriminate between two groups according to an external gold standard [17]. The ROC curve is a probability curve in which each value represents the sensitivity versus 1-specificity for all possible cut-off points. The AUC represents the integral under the ROC curve fitted through these points and serves as a measure of discrimination. A value of 0.5, represented by the diagonal, indicates that the measurement instrument has no discrimination capacity to distinguish between the two groups, and an AUC greater 0.70 is recommended for sufficient responsiveness [17]. In the present study, perceived recovery after three months (PGIC dichotomized) was used as external measure, and data of patients at start and after three months were analyzed.

The statistical analyses were conducted using R (version 3.5.0) for the validation measures and IBM SPSS (version 25) for the remainder. The significance level alpha was set at 0.05.

## Results

Data was collected between January 2017 and February 2019. In total, 240 participants (166 females and 74 males; mean age = 13.05 ± 1.70 years) were included, namely 100 patients recruited in chiropractic practices and 140 pupils in schools. Of the 140 pupils, 109 had never sought medical care because of spinal pain and served as control participants. 31 pupils had previously sought care for spinal pain and were included in the patient group alongside the 100 patients from the chiropractic practices, resulting in 131 individuals in the patient group. At start, all 240 participants filled in the questionnaires. After two weeks, 106 patients (response rate = 81 %; sum score of pain frequency at baseline: responders median = 6, non-responders median = 4) and 85 control participants (response rate = 78 %) answered the questionnaires. After three months, 95 patients (response rate = 73 %; sum score pain of frequency at baseline: responders median = 6, non-responders median = 5) and 101 control participants (response rate = 93 %) could be reached. Detailed information about the study population is presented in Table 1.

**Table 1:** Sociodemographic characteristics of study participants at start, after two weeks and three months

		**Patients**			**Control participants**		
		**Male**	**Female**	**Total**	**Male**	**Female**	**Total**
Start	N	39	92	131 (100 %)	35	74	109 (100 %)
	Age (years), mean (SD)	12.18 (SD 1.37)	13.28 (SD 1.69)	12.95 (SD 1.67)	12.49 (SD 1.69)	13.47 (SD 1.69)	13.16 (SD 1.74)
After 2 weeks	N (%)	28	78	106 (81 %)	26	59	85 (78 %)
	Age (years), mean (SD)	12.32 (SD 1.44)	13.28 (SD 1.71)	13.03 (SD 1.69)	12.27 (SD 1.64)	13.27 (SD 1.59)	12.96 (SD 1.66)
After 3 months	N (%)	21	74	95 (73 %)	34	67	101 (93 %)
	Age (years), mean (SD)	12.10 (SD 1.22)	13.39 (SD 1.70)	13.11 (SD 1.69)	12.53 (SD 1.69)	13.45 (SD 1.66)	13.14 (SD 1.72)

Scores per spinal region and the calculated sum scores are shown in Table 2.

**Table 2:** Scores per spinal region and sum scores of the German version of the Young Spine Questionnaire at start, after 2 weeks and 3 months

**SCORES PER SPINAL REGION**			**Patients (N = 131)**			**Control Participants (N = 109)**		
**Spinal region**	**Variable**	**Answer (score)**	**Start** **N (%)**	**2 weeks** **N (%)**	**3 months** **N (%)**	**Start** **N (%)**	**2 weeks** **N (%)**	**3 months** **N (%)**
Neck	Pain frequency	Never (0)	8 (6.1)	9 (8.5)	4 (4.2)	12 (11.0)	11 (12.9)	15 (14.9)
		Yes, once or twice (1)	30 (22.9)	19 (17.9)	27 (28.4)	51 (46.8)	38 (44.7)	50 (49.5)
		Yes, once in a while (2)	49 (37.4)	36 (34.0)	36 (37.9)	37 (33.9)	33 (38.8)	29 (28.7)
		Yes, often (3)	44 (33.6)	42 (39.6)	28 (29.5)	9 (8.3)	3 (3.5)	7 (6.9)
	Week prevalence	No (0)	58 (44.3)	46 (43.4)	48 (50.5)	86 (78.9)	61 (71.8)	70 (69.3)
		Yes (1)	73 (55.7)	60 (56.6)	47 (49.5)	23 (21.1)	24 (28.2)	31 (30.7)
	Point prevalence	No (0)	92 (70.2)	71 (67.0)	76 (80.0)	104 (96.3)	77 (90.6)	95 (94.1)
		Yes (1)	39 (29.8)	35 (33.0)	19 (20.0)	4 (3.7)	8 (9.4)	6 (5.9)
	Pain intensity	No pain (0)	11 (8.4)	9 (8.5)	8 (8.4)	16 (14.7)	14 (16.5)	16 (15.8)
		1	18 (22.1)	12 (11.3)	16 (16.8)	32 (29.4)	28 (32.9)	42 (41.6)
		2	29 (22.1)	28 (26.4)	25 (26.3)	38 (34.9)	20 (23.5)	24 (23.8)
		3	34 (26.0)	28 (26.4)	21 (22.1)	17 (15.6)	18 (21.2)	16 (15.8)
		4	26 (19.8)	21 (19.8)	17 (17.9)	6 (5.5)	4 (3.7)	3 (3.0)
		Very much pain (5)	13 (9.9)	8 (7.5)	8 (8.4)	0 (0)	1 (0.9)	0 (0)
Mid back	Pain frequency	Never (0)	24 (18.5)	20 (18.9)	16 (16.8)	33 (30.3)	30 (35.3)	36 (35.6)
		Yes, once or twice (1)	40 (30.8)	25 (23.6)	30 (31.6)	42 (38.5)	35 (41.2)	42 (41.6)
		Yes, once in a while (2)	32 (24.6)	34 (32.1)	34 (35.8)	27 (24.8)	16 (18.8)	19 (18.8)
		Yes, often (3)	34 (26.2)	27 (25.5)	15 (5.8)	7 (6.4)	4 (4.7)	4 (4.0)
	Week prevalence	No (0)	70 (53.8)	61 (57.5)	66 (69.5)	83 (76.1)	65 (77.4)	84 (83.2)
		Yes (1)	60 (46.2)	45 (42.5)	29 (30.5)	26 (23.9)	19 (22.6)	17 (16.8)
	Point prevalence	No (0)	103 (79.2)	83 (79.0)	82 (86.3)	102 (94.4)	78 (91.8)	95 (94.1)
		Yes (1)	27 (20.8)	22 (21.0)	13 (13.7)	6 (5.6)	7 (8.2)	6 (5.9)
	Pain intensity	No pain (0)	27 (20.8)	22 (20.8)	20 (20.8)	36 (33.0)	33 (38.8)	37 (36.6)
		1	13 (10.0)	15 (14.2)	17 (17.7)	30 (27.5)	18 (21.2)	29 (28.7)
		2	31 (23.8)	25 (23.6)	21 (21.9)	19 (17.4)	20 (23.5)	19 (18.8)
		3	28 (21.5)	25 (23.6)	22 (22.9)	18 (16.5)	8 (9.4)	11 (10.9)
		4	23 (17.7)	11 (10.4)	11 (11.5)	5 (4.6)	5 (5.9)	2 (2.0)
		Very much pain (5)	8 (6.2)	8 (7.5)	5 (5.2)	1 (0.9)	1 (1.2)	3 (3.0)
Low back	Pain frequency	Never (0)	21 (16.0)	11 (10.4)	12 (12.6)	44 (40.4)	35 (41.2)	34 (33.7)
		Yes, once or twice (1)	38 (29.0)	20 (18.9)	29 (30.5)	34 (31.2)	30 (35.3)	40 (39.6)
		Yes, once in a while (2)	24 (18.3)	32 (30.2)	21 (22.1)	21 (19.3)	12 (14.1)	19 (18.8)
		Yes, often (3)	48 (36.6)	43 (40.6)	33 (34.7)	10 (9.2)	8 (9.4)	8 (7.9)
	Week prevalence	No (0)	70 (53.4)	53 (50.0)	50 (52.6)	90 (82.6)	71 (83.5)	82 (81.2)
		Yes (1)	61 (46.6)	53 (50.0)	45 (47.4)	19 (17.4)	14 (16.5)	19 (18.8)
	Point prevalence	No (0)	98 (75.4)	72 (67.9)	75 (78.9)	101 (92.7)	83 (97.6)	93 (93.0)
		Yes (1)	32 (24.6)	34 (32.1)	20 (21.1)	8 (7.3)	2 (2.4)	7 (7.0)
	Pain intensity	No pain (0)	26 (19.8)	15 (14.2)	14 (14.7)	47 (43.1)	39 (45.9)	36 (35.6)
		1	19 (14.5)	12 (11.3)	9 (9.5)	25 (22.9)	15 (17.6)	21 (20.8)
		2	29 (22.1)	15 (14.2)	27 (28.4)	18 (16.5)	15 (17.6)	34 (33.7)
		3	20 (15.3)	25 (23.6)	18 (18.9)	14 (12.8)	12 (14.1)	6 (5.9)
		4	18 (13.7)	29 (27.4)	18 (18.9)	4 (3.7)	2 (2.4)	2 (2.0)
		Very much pain (5)	19 (14.5)	10 (9.4)	9 (9.5)	1 (0.9)	2 (2.4)	2 (2.0)
**SUM SCORES**			**Patients (N = 131)**			**Control Participants (N = 109)**		
**Variable**		**Range**	**Start** **Median (IQR)**	**2 weeks** **Median (IQR)**	**3 months** **Median (IQR)**	**Start** **Median (IQR)**	**2 weeks** **Median (IQR)**	**3 months** **Median** **(IQR)**
Sum	Pain frequency	0–9	6 (3)	6 (3)	5 (3)	3 (3)	3 (3)	3 (2)
	Week prevalence	0–3	2 (1)	2 (1.5)	1 (2)	0 (1)	0 (1)	0 (1)
	Point prevalence	0–3	0 (1)	1 (1)	0.5 (1)	0 (0)	0 (0)	0 (0)
	Pain intensity	0–15	7 (4)	8 (5)	7 (3.25)	4 (4)	4 (5)	3.94 (2.83)

IQR = interquartile range

### Validity

Patients reported significantly higher sum scores of pain frequency (*p* < 0.001), week prevalence (*p* < 0.001), point prevalence (*p* < 0.001) and pain intensity (*p* < 0.001) compared to the control participants (Table 2). Control participants older than 12 years had significantly higher sum scores of pain frequency, week prevalence and pain intensity compared to the younger controls, indicating that the G-YSQ captures the natural age-related increase in spinal pain prevalence [3, 4]. In contrast, older and younger control participants did not significantly differ in point prevalence (Table 3).

**Table 3:** Comparison of sum scores of the G-YSQ between control participants older than 12 years and control participants aged 12 or younger

Variable		Range	Control Participants ≤ 12 years (*N* = 72) Median (IQR)	Control Participants > 12 years (*N* = 37) Median (IQR)	p-value
Sum	Pain frequency	0–9	4 (4)	5 (3)	0.001
	Week prevalence	0–3	1(1)	1 (2)	0.009
	Point prevalence	0–3	0 (1)	0 (1)	0.931
	Pain intensity	0–15	5 (4)	6 (5)	0.002

IQR = interquartile range

Significantly fewer patients than controls reported excellent general health (*p* < 0.001) (Table 4).

**Table 4:** Self-rated general health in the two cohorts

	Patients	Control participants
**Self-rated general health**	**N = 131 (%)**	**N = 108 (%)**
Excellent	11 (8.4)	30 (27.8)
Very good	53 (40.5)	45 (41.7)
Good	60 (45.8)	27 (25.0)
Fair	6 (4.6)	5 (4.6)
Poor	1 (0.8)	1 (0.9)

**Fig. 2:**
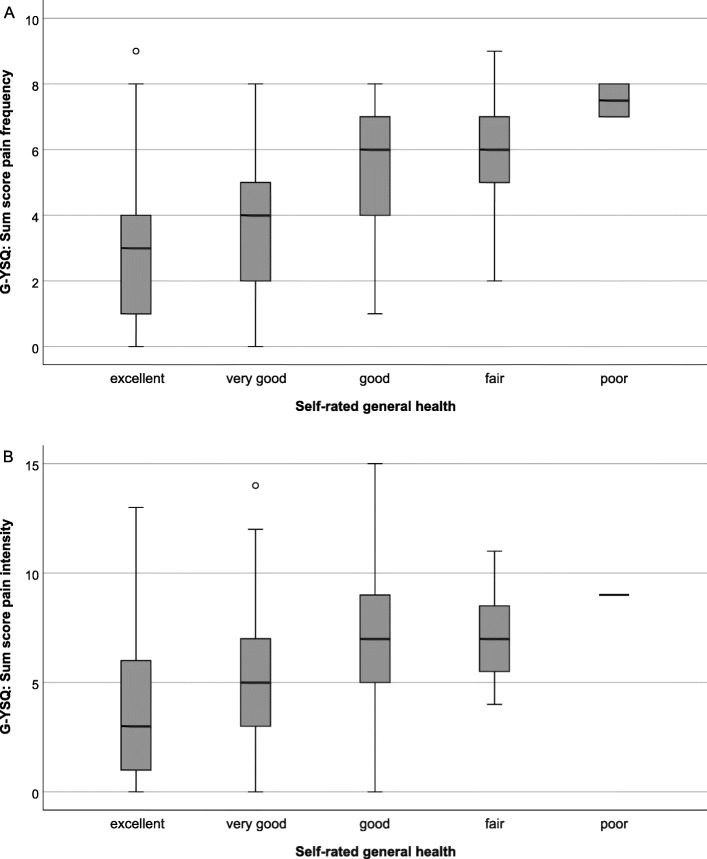
Pain frequency sum score (**A**) and pain intensity sum score (**B**) in the five categories of self-rated general health

Lower self-ratings of general health were associated with higher sum scores in the G-YSQ (Fig. 2): the correlations between self-rated general health and the pain frequency sum score (r_s_=0.46), the pain prevalence sum scores (week prevalence: r_s_=0.42; point prevalence: r_s_=0.28) and the pain intensity sum score (r_s_=0.39) were moderate to fair.

**Fig. 3:**
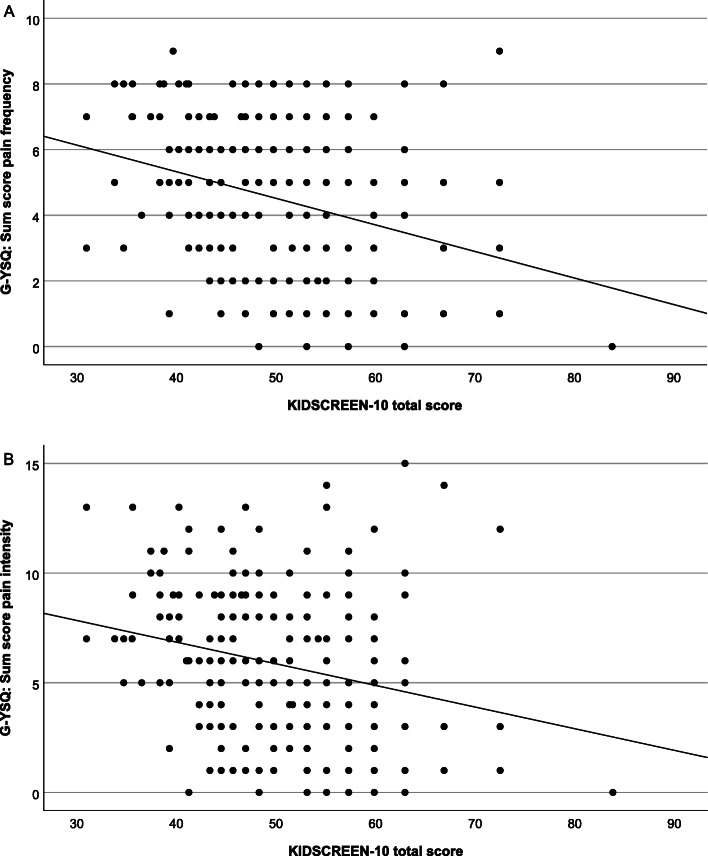
Scatter plots of pain frequency sum score (**A**) and pain intensity sum score (**B**) against KIDSCREEN-10 total score

The two groups showed comparable KIDSCREEN-10 total scores [control participants 50.56+/-9.27; patients 50.24+/-8.33; t(233) = 0.28, *p* = 0.778], and the KIDSCREEN-10 total scores were fairly correlated with the sum scores of week prevalence (r_s_=-0.30) and point prevalence (r_s_=-0.20) (Fig. 3).

The pain severity subgroups significantly differed in the KIDSCREEN-10 sum score [F(4,230) = 7.26, *p* < 0.001] and in the self-rating of general health [H(4) = 51.94, *p* < 0.001]. Post-hoc tests indicated that the ‘no pain’ and the ‘one-sited moderate pain’ groups reported significantly higher KIDSCREEN-10 sum scores and higher ratings of general health than the ‘one-sited severe pain’ and the ‘multiple severe pain’ groups (p-values for sum score = 0.001 and 0.005; p-values for general health < 0.001). The ‘multiple moderate pain’ subgroup did not significantly differ from any of the other pain groups in terms of the KIDSCREEN-10 sum score, but rated general health significantly better than the ‘multiple severe pain’ subgroup (*p* = 0.021): e.g. 28 % of the participants in the ‘multiple moderate pain’ subgroup, compared to 5 % in the ‘multiple severe pain’ subgroup, rated their health as excellent (Table 5).

**Table 5:** Health-related quality of life in the five pain severity subgroups

Pain severity subgroup	KIDSCREEN-10		Self-rated general health					
	N	**Score (± SD)**	**N**	**Excellent** **(%)**	**Very good (%)**	**Good (%)**	**Fair (%)**	**Poor (%)**
No pain	41	54.55 (± 9.19)	42	17 (40.5)	18 (42.9)	7 (16.7)	0 (0)	0 (0)
One-sited moderate pain	32	54.28 (± 8.49)	32	9 (28.1)	20 (62.5)	2 (6.3)	1 (3.1)	0 (0)
Multiple moderate pain	31	51.25 (± 8.59)	32	9 (28.1)	11 (34.4)	9 (28.1)	3 (9.4)	0 (0)
One-sited severe pain	73	48.02 (± 7.84)	73	3 (4.1)	32 (43.8)	35 (47.9)	3 (4.1)	0 (0)
Multiple severe pain	58	47.82 (± 8.03)	60	3 (5.0)	17 (28.3)	34 (56.7)	4 (6.7)	2 (3.3)

SD = standard deviation

Thus, although the correlations with the KIDSCREEN-10 sum score and the self-rated general health were lower than expected, all hypotheses could be confirmed, which indicates good construct validity of the G-YSQ.

### Reliability

The G-YSQ showed good reliability in terms of pain intensity and pain frequency (Table 6). No pain or stable pain during the past two weeks was reported by 83 control participants, and the reliability of week prevalence in those data was moderate for all spinal regions (the neck, the middle and the lower back) as well as for the sum score. Reliability of point prevalence was fair to moderate for the neck, the middle and the lower back, and moderate for the sum score. The reliability of the questions on the consequences of spinal pain was good for school absence and doctor visits and moderate for restriction in sports. All questions on parental spinal pain showed moderate reliability (Table 6).

**Table 6:** Reliability of all domains of the German version of the Young Spine Questionnaire

**Pain intensity**	Neck	ICC_(3,1)_ = 0.80
	Mid back	ICC_(3,1)_ = 0.77
	Low back	ICC_(3,1)_ = 0.81
	Sum score	ICC_(3,1)_ = 0.88
**Pain frequency**	Neck	ICC_(3,1)_ = 0.83
	Mid back	ICC_(3,1)_ = 0.77
	Low back	ICC_(3,1)_ = 0.80
	Sum score	ICC_(3,1)_ = 0.88
**Week prevalence**	Neck	к=0.55
	Mid back	к=0.60
	Low back	к=0.48
	Sum score	ICC_(3,1)_ = 0.68
**Point prevalence**	Neck	к=0.42
	Mid back	к=0.25
	Low back	к=0.32
	Sum score	ICC_(3,1)_ = 0.60
**Pain consequences**	School absence	ICC_(3,1)_ = 0.78
	Doctor visits	ICC_(3,1)_ = 0.79
	Restriction in sports	ICC_(3,1)_ = 0.74
**Parental back pain**	Back pain father	ICC_(3,1)_ = 0.69
	Back pain mother	ICC_(3,1)_ = 0.60
	Work interference father	ICC_(3,1)_ = 0.71
	Work interference mother	ICC_(3,1)_ = 0.73

### Responsiveness

**Fig. 4:**
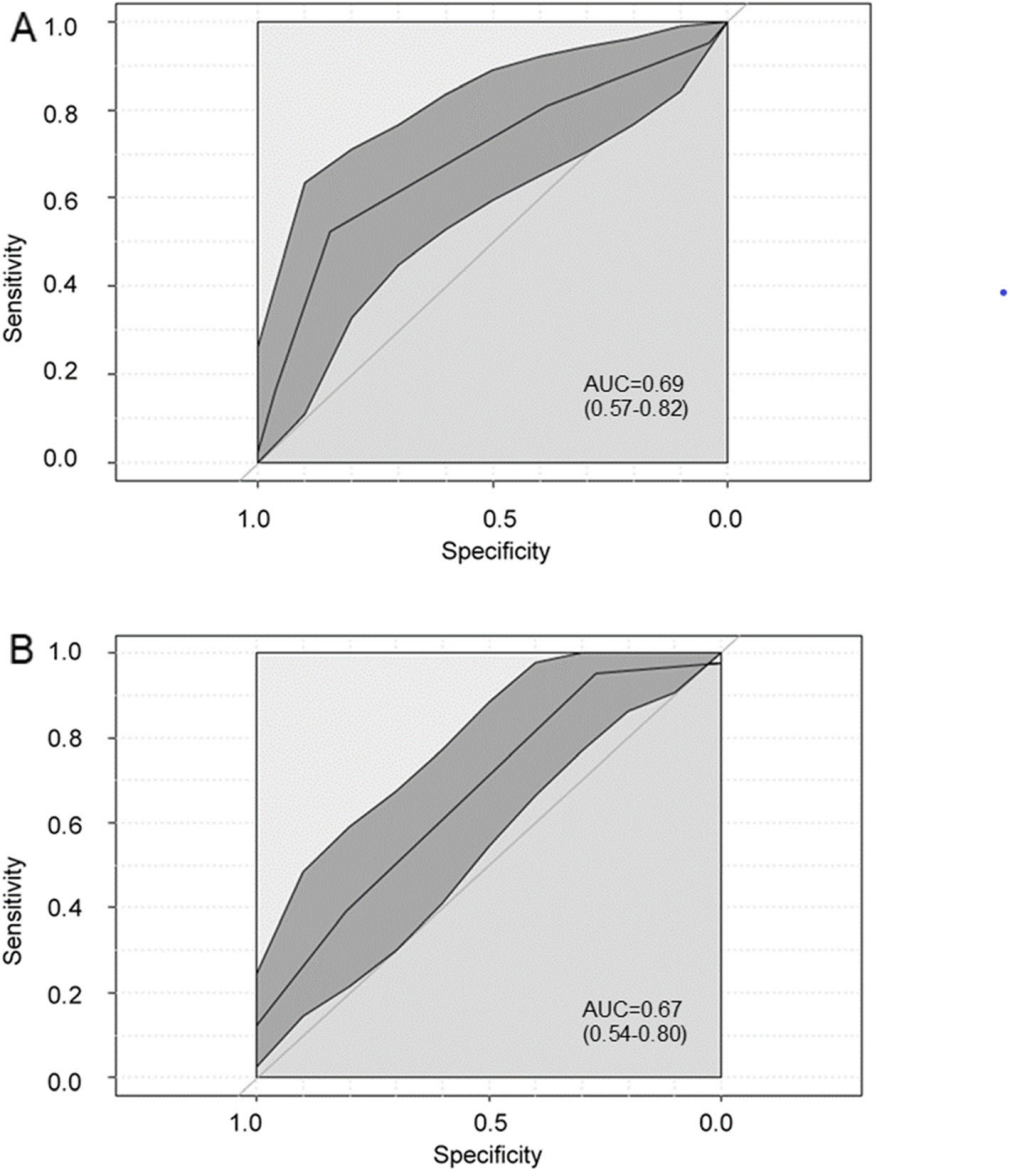
Receiver operating characteristic **(**ROC) curve and corresponding area under the curve (AUC) for week prevalence (**A**) and point prevalence (**B**). Confidence interval of AUC is shown in brackets

Of the 68 patients who answered the PGIC after three months (27 patients answered the G-YSQ, but did not fill in the PGIC), 61.8 % reported clinically significant improvement. AUC was 0.69 (95 %CI = 0.57–0.82) for the sum of week prevalence and 0.67 (95 %CI = 0.54–0.80) for the sum of point prevalence (Fig. 4), indicating insufficient responsiveness.

## Discussion

The aims of this study were to translate the YSQ into German according to scientific guidelines and to determine construct validity, test-retest reliability, and responsiveness of the G-YSQ. The translation process of the original YSQ into German was straightforward apart from two minor issues in terms of cross-cultural adaptation. Except for the question on point prevalence, the G-YSQ was shown to possess construct validity and sufficient test-retest reliability, but its responsiveness needs to be improved.

Although the study population of the present study was older than the population for which the original questionnaire was designed, the G-YSQ discriminated well between patients and controls and between older and younger children and adolescents, depicting the natural age-related increase in spinal pain prevalence [3, 4]. The G-YSQ (week and point prevalence) correlated fairly with the KIDSCREEN-10 sum score. This finding might reflect a weaker association between back pain in childhood and adolescence and psychosocial issues than commonly believed [27–32] because eight of the ten questions of the KIDSCREEN-10 are on psychosocial topics and only two on physical health. Self-rated general health was better in the control group. However, the correlation to pain frequency, pain intensity and week prevalence was fair and weaker than hypothesized, which might reflect that the majority of children and adolescents, approximately 9 out of 10 [33], are not seriously impacted by their back pain [33, 34]. Nevertheless, a minority is and identifying this minority, i.e. differentiating between trivial and consequential back pain, is one of the biggest challenges with respect to back pain in childhood and adolescence [35]. Although most spinal pain in childhood and adolescence is self-limiting [36] or ‘trivial’ [35], pain problems in children and adolescents in general [37], and back pain [38] and LBP [33] in particular, are associated with lower health-related quality of life. A decline in quality of life might be an early indicator of potential vulnerability during development [39] and might be a symptom of a multidimensional process [33], possibly leading to consequences in adulthood (‘consequential’ pain [35]). To avoid medicalization of the problem and to target treatment, consequential back pain should be differentiated from trivial back pain in childhood and adolescence [35]. In the present study, the cut-off for potentially consequential spinal pain as defined by a significant impact on health-related quality of life was having severe pain (one-sited or multiple), and 32 % of the controls belonged to these subgroups. The present study shows that both, pain frequency and intensity, need to be assessed in each spinal region to capture all children and adolescents whose spinal pain impacts their quality of life and might therefore be consequential. Whether these individuals will indeed develop a back problem in adulthood needs to be verified in longitudinal studies.

The G-YSQ also showed test-retest reliability, particularly for pain intensity and pain frequency. The lower values for validity and reliability of the question on point prevalence might reflect the natural clinical course of spinal pain in childhood and adolescence, which appears to fluctuate [40], rather than a deficiency of the assessment tool. Nevertheless, regarding moderate reliability, deficiencies in construct validity and insufficient responsiveness, the benefit of asking for point prevalence is questionable, and omitting this question is recommended. In its present form, the YSQ/G-YSQ cannot be recommended to be used to measure change. This is not surprising given that the answers to the only questions possibly reflecting change (presence or absence of low back pain, mid back pain or neck pain during the last week) are binary (yes/no). Refining this dichotomous structure similarly to the question on pain frequency (‘often’, ‘once in a while’, ‘once or twice’, ‘never’) might enhance the responsiveness of the YSQ/G-YSQ. Similarly, it might be worth attempting to address the problem of inconsistent answers to the question on pain frequency and corresponding pain intensity by rearranging these items (rFPS for pain intensity directly following the question on pain frequency).

The age range of 10 to 16 years was selected as it appears to be the crucial period for developing spinal pain [3, 4]. Nevertheless, this implies that the findings cannot be generalized to younger children. Pupils who indicated that they have seen a doctor because of back or neck pain (*N* = 31) were analyzed in the group of patients because we considered them as having a back or neck problem. However, these participants did not necessarily have back pain at the time of the survey. We therefore repeated the analysis without these pupils. Results were similar in terms of group differences, ICC- and AUC-values. Group differences became even more pronounced without these pupils. Three adolescents (aged 13 and 15) of the pilot phase reported that they preferred indicating pain intensity on a scale rather than on the rFPS. There is no optimal pain assessment to be used throughout development from childhood to adolescence [41, 42]: for school-aged children (from 8 to 12 years) the rFPS is recommended [42]. Adolescents prefer visual analogue scales (VAS) or numeric rating scales (NRS) [42], although faces scales have been reported to also be well accepted [41, 42]. A supplementation of the rFPS with a VAS or NRS might be useful to make the YSQ/G-YSQ applicable throughout childhood and adolescence. Not all participants could be reached for the follow-up after two weeks and three months. Because data of pupils were collected at schools, these missing values were most likely at random. However, a closer look at differences between responding and non-responding patients revealed that they were comparable in terms of gender and age. There was a tendency for the responders to have more frequent and more intense back or neck pain at start compared to the non-responders. Because only the start data were used for validity assessment and the assessment of reliability and responsiveness involved only intra-subject comparisons, it seems unlikely that data from the non-responders would have changed the findings. Another study limitation might have been that the first re-assessment took place after two weeks, although the question on week prevalence in the YSQ and the questions in the KIDSCREEN-10 refer to the last week. However, there is no standard for an ideal time period for testing reliability, and a time interval of two weeks is commonly used [17].

## Conclusions

The G-YSQ was shown to possess construct validity and sufficient test-retest reliability to assess back pain in children and adolescents between 10 and 16 years of age. To measure responsiveness, the YSQ/G-YSQ has to be adapted, possibly by asking for pain frequency instead of (dichotomous) pain prevalence during the last week. Severe pain, either in one or multiple spinal sites, was associated with reduced health-related quality of life and might be consequential, which needs to be verified in prospective studies from adolescence into adulthood.

## Supplementary information

Additional file 1 English version of the Young Spine Questionnaire. This is the original version of the Young Spine Questionnaire (Lauridsen HH, Hestbaek L. Development of the young spine questionnaire. BMC Musculoskelet Disord. 2013; 14:185)

Additional file 2 German version of the Young Spine Questionnaire. This is the German version of the Young Spine (G-YSQ) that was validated in this study

## Data Availability

The datasets used are available from the corresponding author upon request.

## Abbreviations

AUC: area under the curve
CI: confidence interval
G-YSQ: German version of the Young Spine Questionnaire
HRQoL: Health-related quality of life
ICC: intraclass correlation coefficient
NRS: numeric rating scale
PGIC: Patient global rating of change
rFPS: revised Faces Pain Scale
ROC: receiver operating curve
VAS: Visual Analogue Scale
YSQ: Young Spine Questionnaire
