# Supplementary material for: Translation and validation of the German version of the Young Spine Questionnaire
Source: BMC Pediatr. 2021 Aug 24;21:359. doi: 10.1186/s12887-021-02804-y (PMC8383347; doi:10.1186/s12887-021-02804-y)
Supplement: Supplementary file 1 — English version of the Young Spine Questionnaire. This is the original version of the Young Spine Questionnaire (Lauridsen HH, Hestbaek L. Development of the young spine questionnaire. BMC Musculoskelet Disord. 2013; 14:185) [file 12887_2021_2804_MOESM1_ESM.docx]

**Translation and validation of the German version of the Young Spine Questionnaire**

Luana Nyirö, DCM^1*^, Tobias Potthoff, MSc, DCM^1*^, Mette Hobaek Siegenthaler, DC^1,2^, Fabienne Riner, MSc^1^, Petra Schweinhardt, MD, PhD^1^, Brigitte Wirth, PT, PhD^1,3^

* These authors contributed equally to this study.

^1^ Integrative Spinal Research Group

Department of Chiropractic Medicine

Balgrist University Hospital and University of Zurich

Forchstr. 340

8008 Zurich

Switzerland

^2^ Holbeinpraxis

Holbeinstrasse 65

4051 Basel

Switzerland

^3^ Winterthur Institute of Health Economics

School of Management and Law

University of Applied Sciences

Gertrudstr. 15

8400 Winterthur

Switzerland

**Corresponding author:**

Brigitte Wirth

Winterthur Institute of Health Economics

School of Management and Law

University of Applied Sciences

Gertrudstr. 15

8400 Winterthur

Switzerland

brigitte.wirth@zhaw.ch

+41 58 934 69 17

## Additional file 1: English version of the Young Spine Questionnaire (Lauridsen HH, Hestbaek L. Development of the young spine questionnaire. BMC Musculoskelet Disord. 2013; 14:185)

| **Name______________________________ Class________________**  This questionnaire is related to the spine and neck. Use only one cross (X) to answer each question. If none of the answers are suitable, place your cross by the answer that is best suited. | | | | | | | | | |
| --- | --- | --- | --- | --- | --- | --- | --- | --- | --- |
|  | | | | | |  | | | |
| **1. The neck is shown in the picture** | | | | | |  | | | |
|  | | | | | |  | | | |
|  | | | 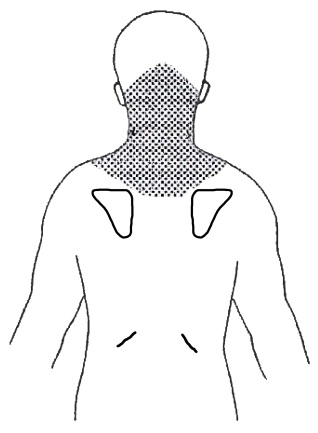  The neck | | | | |  | |
|  | | | Person seen from behind | | | | |  | |
|  | | |  | | | | |  | |
|  | | |  | | | | |  | |
| 1a. Have you had pain in the neck? | | | | | | 🞏 Often  🞏 Once in a while  🞏 Once or twice  🞏 Never | | | |
|  | | | | | |  |  |  |  |
|  | | | | | |  |  |  |  |
|  | | | | | |  | | | |
| 1b. Have you had neck pain in **the last week**? | | | | | | 🞏 Yes  🞏 No | | | |
|  | | | | | |  |  |  |  |
|  | | | | | |  | | | |
| 1c. Have you had neck pain **today**? | | | | | | 🞏 Yes  🞏 No | | | |
|  | | | | | |  |  |  |  |
|  | | | | | |  | | | |
| The faces below show how much something can hurt. The pain ranges from ‘No pain’ to ‘A lot of pain’. | | | | | | | | | |
|  | | | |  | | | | | |
| 1d. Put a cross (X) on the face which shows how much pain you have had in the neck when it was worst. | | | | | | | | | |
|  | | | | |  | | | | |
| No pain |  |  | | |  | |  | | A lot of pain |
| 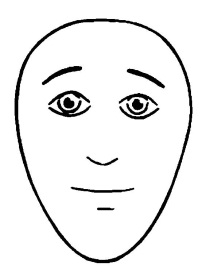 | 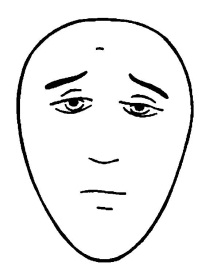 | 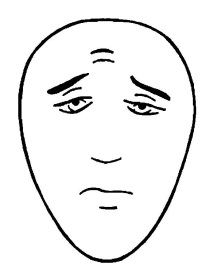 | | | 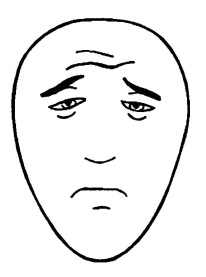 | | 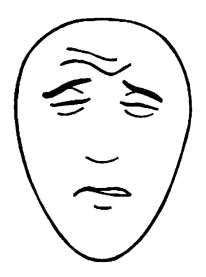 | | 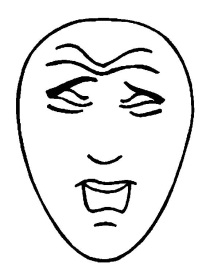 |

| **2. The middle of the back is shown in the picture.** | | | |  | | |
| --- | --- | --- | --- | --- | --- | --- |
|  | | | |  | | |
|  | | 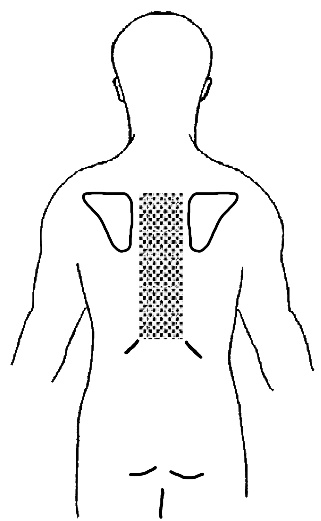  Middle of the back | | |  | |
|  | | Person seen from behind | | |  | |
|  | | | |  | | |
| 2a. Have you had pain in the middle of the back? | | | | 🞏 Often  🞏 Once in a while  🞏 Once or twice  🞏 Never | | |
|  | | | |  |  |  |
|  | | | |  |  |  |
|  | | | |  | | |
| 2b. Have you had pain in the middle of the back in **the last week**? | | | | 🞏 Yes  🞏 No | | |
|  | | | |  | | |
| 2c. Have you had pain in the middle of the back **today**? | | | | 🞏 Yes  🞏 No | | |
|  | | | |  |  |  |
|  | | | |  | | |
| 2d. Put a cross (X) on the face which shows how much pain you have had in the middle of the back when it was worst. | | | | | | |
|  | | | |  | | |
| No pain |  |  |  | |  | A lot of pain |
| 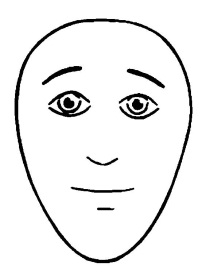 | 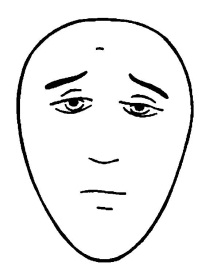 | 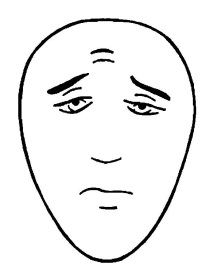 | 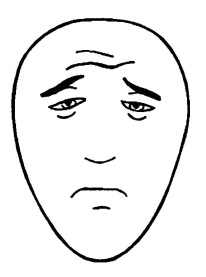 | | 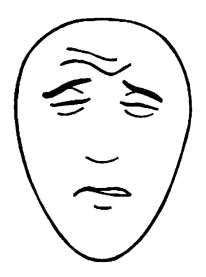 | 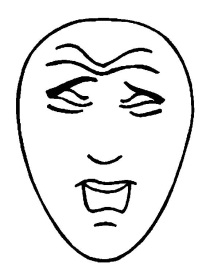 |

| **3. The lower back is shown in the picture.** | | | | |  | | | |
| --- | --- | --- | --- | --- | --- | --- | --- | --- |
|  | | | | |  | | | |
|  | | | 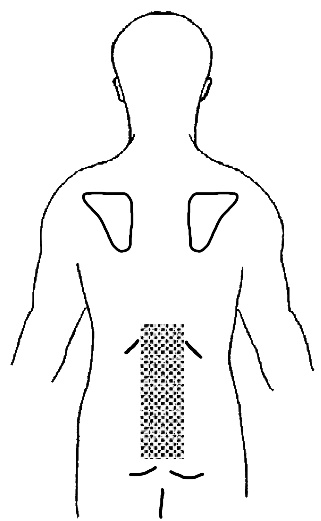  The lower back | | | |  | |
|  | | | Person seen from behind | | | |  | |
|  | | | | |  | | | |
| 3a. Have you had pain in the lower back? | | | | | 🞏 Often  🞏 Once in a while  🞏 Once or twice  🞏 Never | | | |
|  | | | | |  |  |  |  |
|  | | | | |  |  |  |  |
|  | | | | |  | | | |
| 3b. Have you had pain in the lower back in **the last week**? | | | | | 🞏 Yes  🞏 No | | | |
|  | | | | |  |  |  |  |
|  | | | | |  | | | |
| 3c. Have you had pain in the lower back **today**? | | | | | 🞏 Yes  🞏 No | | | |
|  | | | | |  |  |  |  |
|  | | | | | | | | |
| 3d. Put a cross (X) on the face which shows how much pain you have had in the lower back when it was worst. | | | | | | | | |
|  | | | |  | | | | |
| No pain |  |  | |  | |  | | A lot of pain |
| 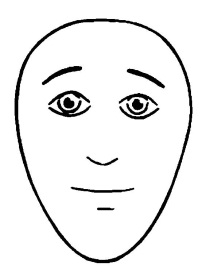 | 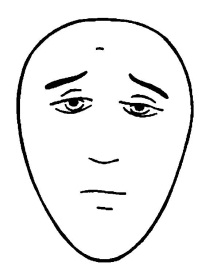 | 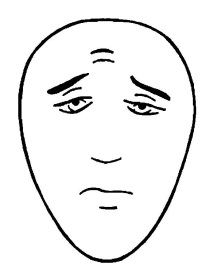 | | 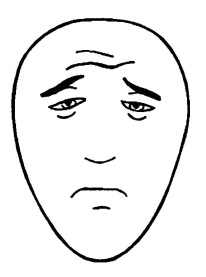 | | 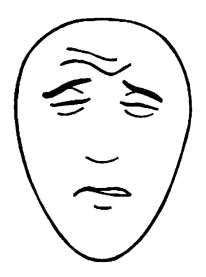 | | 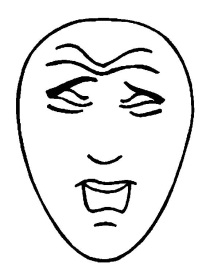 |

| **4. School, recreation and treatment** |  | |  |
| --- | --- | --- | --- |
|  |  | |  |
| 4a. Have you stayed home from school because of neck or back pain? | | 🞏 Often  🞏 Once in a while  🞏 Once or twice  🞏 Never | |
|  | |  |  |
|  | |  |  |
|  | |  | |
| 4b. Has neck or back pain sometimes stopped you from doing sports? | | 🞏 Often  🞏 Once in a while  🞏 Once or twice  🞏 Never | |
|  | |  | |
| 4c. Have you been to a doctor, chiropractor or physiotherapist because of neck or back pain? | | 🞏 Often  🞏 Once in a while  🞏 Once or twice  🞏 Never | |
|  | |  |  |
|  | |  |  |
| **5. The family** | |  |  |
|  | |  |  |
| 5a Has your **father** or stepfather ever had back or neck pain? | | 🞏 Yes  🞏 No |  |
|  | |  |  |
| 5b. If he has, has it kept him home from work? | | 🞏 Often  🞏 Once in a while  🞏 Never | |
|  | |  |  |
|  | |  |  |
| 5c Has your **mother** or stepmother ever had back or neck pain? | | 🞏 Yes  🞏 No |  |
|  | |  |  |
| 5d. If she has, has it kept her home from work? | | 🞏 Often  🞏 Once in a while  🞏 Never | |
|  | |  |  |
