# Supplementary material for: Translation and validation of the German version of the Young Spine Questionnaire
Source: BMC Pediatr. 2021 Aug 24;21:359. doi: 10.1186/s12887-021-02804-y (PMC8383347; doi:10.1186/s12887-021-02804-y)
Supplement: Supplementary file 2 — German version of the Young Spine Questionnaire. This is the German version of the Young Spine (G-YSQ) that was validated in this study [file 12887_2021_2804_MOESM2_ESM.docx]

**Translation and validation of the German version of the Young Spine Questionnaire**

Luana Nyirö, DCM^1*^, Tobias Potthoff, MSc, DCM^1*^, Mette Hobaek Siegenthaler, DC^1,2^, Fabienne Riner, MSc^1^, Petra Schweinhardt, MD, PhD^1^, Brigitte Wirth, PT, PhD^1,3^

* These authors contributed equally to this study.

^1^ Integrative Spinal Research Group

Department of Chiropractic Medicine

Balgrist University Hospital and University of Zurich

Forchstr. 340

8008 Zurich

Switzerland

^2^ Holbeinpraxis

Holbeinstrasse 65

4051 Basel

Switzerland

^3^ Winterthur Institute of Health Economics

School of Management and Law

University of Applied Sciences

Gertrudstr. 15

8400 Winterthur

Switzerland

**Corresponding author:**

Brigitte Wirth

Winterthur Institute of Health Economics

School of Management and Law

University of Applied Sciences

Gertrudstr. 15

8400 Winterthur

Switzerland

brigitte.wirth@zhaw.ch

+41 58 934 69 17

**Additional file 2:** German version of the Young Spine Questionnaire

| **Klasse: Alter:**  **Geschlecht (bitte Zutreffendes unterstreichen): Knabe / Mädchen**  Dieser Fragebogen bezieht sich auf den Rücken und den Nacken. Beantworte jede Frage mit nur einem Kreuz (X). Wenn keine Antwort passt, kreuze die Antwort an, die am ehesten zutrifft.  An die Mädchen: Im Folgenden wird nur nach Rückenschmerzen gefragt, welche **nicht** im Zusammenhang mit deiner Monatsblutung (Menstruation/Periode) auftreten.  **1. Dieses Bild zeigt den Nacken:** | | | | | | | | |
| --- | --- | --- | --- | --- | --- | --- | --- | --- |
|  | | | 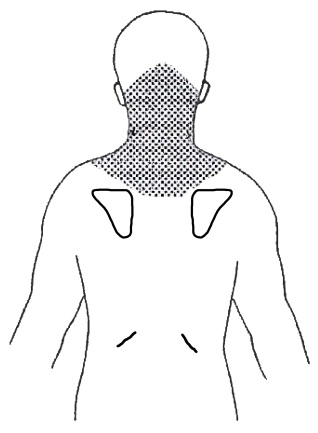  Nacken | | | |  | |
|  | | | Mensch von hinten | | | |  | |
|  | | |  | | | |  | |
|  | | |  | | | |  | |
| 1a. Hattest du schon Schmerzen im Nacken? | | | | | 🞏 Ja, schon oft  🞏 Ja, ab und zu  🞏 Ja, ein- oder zweimal  🞏 Nein, noch nie | | | |
|  | | | | |  |  |  |  |
|  | | | | |  |  |  |  |
|  | | | | |  | | | |
| 1b. Hattest du **in der letzten Woche** Schmerzen im Nacken? | | | | | 🞏 Ja  🞏 Nein | | | |
|  | | | | |  |  |  |  |
|  | | | | |  | | | |
| 1c. Hast du **heute** Schmerzen im Nacken? | | | | | 🞏 Ja  🞏 Nein | | | |
|  | | | | |  |  |  |  |
|  | | | | |  | | | |
| Die folgenden Gesichter zeigen, wie stark etwas wehtun kann. Es geht von ‚keine Schmerzen‘ bis ‚sehr starke Schmerzen‘. | | | | | | | | |
| 1d. Mach ein Kreuz (X) auf das Gesicht, das zeigt, wie stark deine Schmerzen im Nacken waren, als sie am schlimmsten waren. | | | | | | | | |
| keine Schmerzen |  |  | |  | |  | | sehr starke Schmerzen |
| 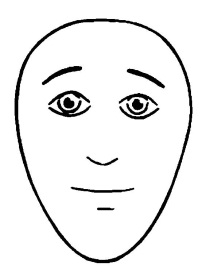 | 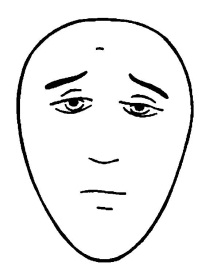 | 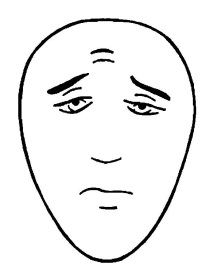 | | 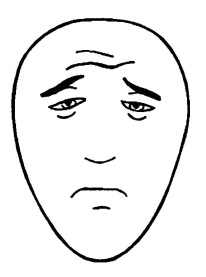 | | 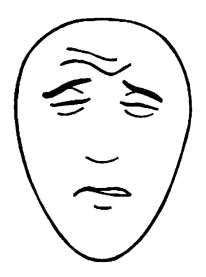 | | 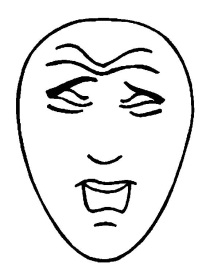 |

| **2. Dieses Bild zeigt den mittleren Rücken:** | | | |  | | |
| --- | --- | --- | --- | --- | --- | --- |
|  | | | |  | | |
|  | | 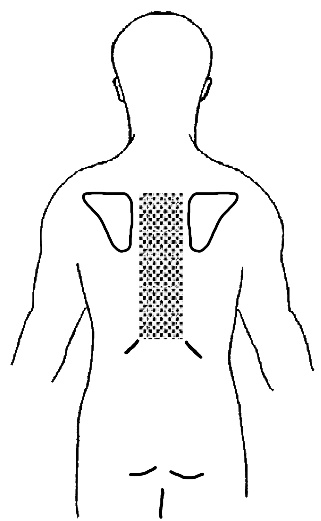  mittlerer Rücken | | |  | |
|  | | Mensch von hinten | | |  | |
|  | | | |  | | |
| 2a. Hattest du schon Schmerzen im mittleren Rücken? | | | | 🞏 Ja, schon oft  🞏 Ja, ab und zu  🞏 Ja, ein- oder zweimal  🞏 Nein, noch nie | | |
|  | | | |  |  |  |
|  | | | |  |  |  |
|  | | | |  | | |
| 2b. Hattest du **in der letzten Woche** Schmerzen im mittleren Rücken? | | | | 🞏 Ja  🞏 Nein | | |
|  | | | |  | | |
| 2c. Hast du **heute** Schmerzen im mittleren Rücken? | | | | 🞏 Ja  🞏 Nein | | |
|  | | | |  |  |  |
| 2d. Mach ein Kreuz (X) auf das Gesicht, das zeigt, wie stark deine Schmerzen im mittleren Rücken waren, als sie am schlimmsten waren. | | | | | | |
| keine Schmerzen |  |  |  | |  | sehr starke Schmerzen |
| 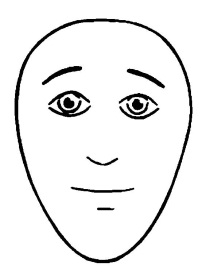 | 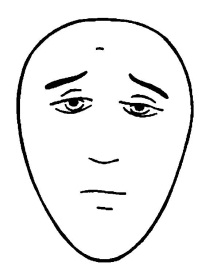 | 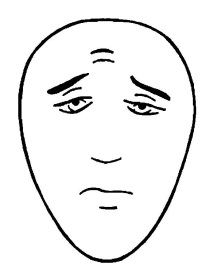 | 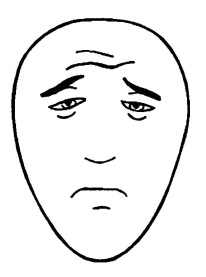 | | 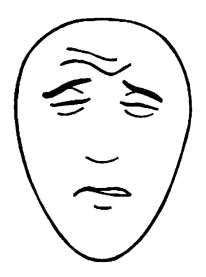 | 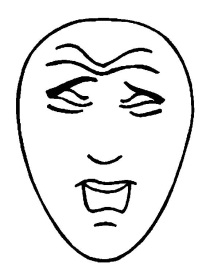 |

| **3. Dieses Bild zeigt den unteren Rücken:** | | | | |  | | | |
| --- | --- | --- | --- | --- | --- | --- | --- | --- |
|  | | | | |  | | | |
|  | | | 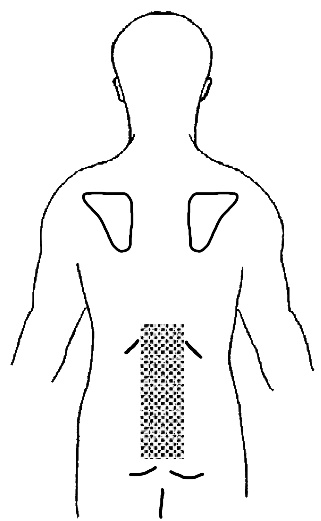  unterer Rücken | | | |  | |
|  | | | Mensch von hinten | | | |  | |
|  | | | | |  | | | |
| 3a. Hattest du schon Schmerzen im unteren Rücken? | | | | | 🞏 Ja, schon oft  🞏 Ja, ab und zu  🞏 Ja, ein- oder zweimal  🞏 Nein, noch nie | | | |
|  | | | | |  |  |  |  |
|  | | | | |  |  |  |  |
|  | | | | |  | | | |
| 3b. Hattest du **in der letzten Woche** Schmerzen im unteren Rücken? | | | | | 🞏 Ja  🞏 Nein | | | |
|  | | | | |  |  |  |  |
|  | | | | |  | | | |
| 3c. Hast du **heute** Schmerzen im unteren Rücken? | | | | | 🞏 Ja  🞏 Nein | | | |
|  | | | | |  |  |  |  |
|  | | | | | | | | |
|  | | | | | | | | |
| 3d. Mach ein Kreuz (X) auf das Gesicht, das zeigt, wie stark deine Schmerzen im unteren Rücken waren, als sie am schlimmsten waren. | | | | | | | | |
| keine Schmerzen |  |  | |  | |  | | sehr starke Schmerzen |
| 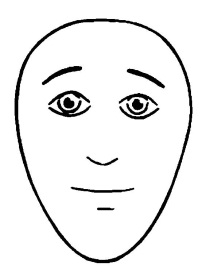 | 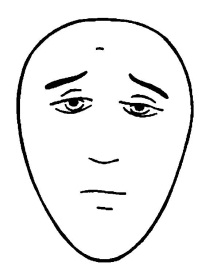 | 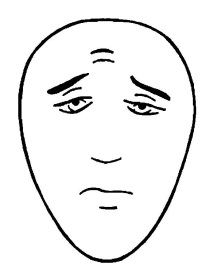 | | 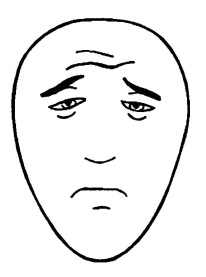 | | 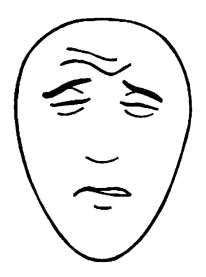 | | 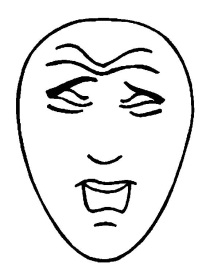 |

| **4. Schule, Freizeit und Behandlung** |  | |  |
| --- | --- | --- | --- |
|  |  | |  |
| 4a. Bist du wegen Schmerzen im Nacken oder Rücken schon zuhause geblieben und nicht zur Schule gegangen? | | 🞏 Ja, schon oft  🞏 Ja, ab und zu  🞏 Ja, ein- oder zweimal  🞏 Nein, noch nie | |
|  | |  |  |
|  | |  |  |
|  | |  | |
| 4b. Konntest du wegen Schmerzen im Nacken oder Rücken schon keinen Sport treiben? | | 🞏 Ja, schon oft  🞏 Ja, ab und zu  🞏 Ja, ein- oder zweimal  🞏 Nein, noch nie | |
| 4c. Warst du wegen Schmerzen im Nacken oder Rücken schon beim Doktor? | | 🞏 Ja, schon oft  🞏 Ja, ab und zu  🞏 Ja, ein- oder zweimal  🞏 Nein, noch nie | |
|  | |  |  |
|  | |  |  |
| **5. Familie** | |  |  |
|  | |  |  |
| 5a. Hatte dein Vater jemals Schmerzen im Nacken oder Rücken? | | 🞏 Ja  🞏 Nein |  |
|  | |  |  |
| 5b. Falls ja, ist er wegen diesen Schmerzen zuhause geblieben und nicht zur Arbeit gegangen? | | 🞏 Ja, schon oft  🞏 Ja, ab und zu  🞏 Nein, noch nie | |
|  | |  |  |
|  | |  |  |
| 5c. Hatte deine Mutter jemals Schmerzen im Nacken oder Rücken? | | 🞏 Ja  🞏 Nein |  |
|  | |  |  |
| 5d. Falls ja, ist sie wegen diesen Schmerzen zuhause geblieben und nicht zur Arbeit gegangen? | | 🞏 Ja, schon oft  🞏 Ja, ab und zu  🞏 Nein, noch nie | |

|  |  |
| --- | --- |
